# The contribution of Specialist Family Physicians to South Africa’s private sector: A position statement

**DOI:** 10.4102/safp.v66i1.6022

**Published:** 2024-10-02

**Authors:** Gail L. Ashford, Sheena Mathew, Gareth A. Fray, Idowu Olusola Irinoye, Andrew Ross, Klaus von Pressentin, Robert Mash

**Affiliations:** 1 Private Practice, Wits Donald Gordon Medical Centre, Johannesburg, South Africa; 2 Department of Obstetrics and Gynecology, Chris Hani Baragwanath Academic Hospital, Johannesburg, South Africa; 3 Specialist Family Physicians in Private, South African Society of Specialist Family Physicians, Johannesburg, South Africa; 4 Division of Family Medicine, Department of Family, Community and Emergency Care, Faculty of Health Sciences, University of Cape Town, Cape Town, South Africa; 5 Private Practice, Cape Town, South Africa; 6 Specialist Family Physicians in Private, South African Academy of Family Physicians, Cape Town, South Africa; 7 Private Practice, East London, South Africa; 8 Specialist Family Physicians in Private, South African Academy of Family Physicians, East London, South Africa; 9 Private Practice, Pretoria, South Africa; 10 South African Society of Specialist Family Physicians (SASOSFP), Pretoria, South Africa; 11 Specialist Family Physicians in Private, South African Academy of Family Physicians, Pretoria, South Africa; 12 Department of Family Medicine, College of Health Sciences, University of KwaZulu-Natal, Durban, South Africa; 13 South African Academy of Family Physicians, Durban, South Africa; 14 Division of Family Medicine and Primary Care, Faculty of Medicine and Health Sciences, Stellenbosch University, Cape Town, South Africa

The South African Academy of Family Physicians (SAAFP) is committed to advocating for the recognition of specialist family physician roles and contributions in the South African private sector. The SAAFP’s mission includes fostering the relationship between family physicians and health facilities, public and private institutions, government authorities, healthcare funders, the public and the medical profession in general. The SAAFP includes private practice sector representatives in its national council and board of directors.

South African Academy of Family Physicians, in collaboration with the South African Society of Specialist Family Physicians (SASOSFP), has written a position statement to clarify the contribution of Specialist Family Physicians (SFP) to the private sector and to advocate for recognition and remuneration appropriate to the speciality. A separate position statement assesses the contribution of SFP to the public sector in South Africa (SA).^1^

The Health Professional Council of SA (HPCSA) recognises the qualification and extended skills set of an SFP, which was gazetted in 2007. Since 2008, SFPs have been trained as expert generalists via accredited registrar posts to strengthen primary healthcare and district health services. By 2019, there were 969 SFPs on the HPCSA register, with an estimated 71% (688 SFPs) in the private sector.^2^ A recent survey among family medicine specialist graduates since 2008 found that 14.5% entered the private sector and an additional 9.6% work in both sectors.^3^

Despite this recognition by the HPCSA, most SFPs working in the private sector are compelled to work as general practitioners (GPs) and cannot order investigations, imaging and medications appropriate to their specialist training. In addition, they are reimbursed according to medical scheme tariffs for GPs and not as specialists. In contrast, the salaries of SFPs in the public sector are on par with those of other specialists. Furthermore, some private hospital groups and funders deny SFPs hospital admission rights, limiting their ability to function as specialists in private hospital settings.

The *Specialist Family Physicians in Private* forum represents the collaboration between SAAFP and SASOSFP and was formed in 2022. It aims to champion and clarify SFPs contribution to the private sector and advocate for recognition, remuneration and specialist privileges appropriate to this consulting discipline. This forum has been engaging with several stakeholders in the private sector to clarify the distinct roles and differing scopes of practice of SFPs, GPs and other specialists (especially internal medicine physicians). Through case studies presented to these stakeholders, the forum has highlighted the under-recognised and untapped potential that a system-wide recognition of SFPs specialist role will bring, such as innovative preventative health packages and tailored managed healthcare packages. In addition to highlighting the value offering of SFPs in realising the core functions of primary care in the private sector, the position statement also emphasises several economic benefits of SFPs. These include:

- *Preventive care:* Focusing on preventive care reduces the need for expensive treatments by identifying health issues early and saving costs associated with advanced medical care.^4^ The SFPs are trained to view their practice as a population at risk and to use motivational interviewing and the behaviour change model to mitigate risk.
- *Reduced hospitalisations*: Regular check-ups and early intervention algorithms (enabled by the patient’s ongoing relationship with their SFP) may reduce hospitalisations. These can prevent health conditions from escalating, decrease healthcare expenses and reduce hospitalisations.^5^
- *Lower overall health costs*: Effectively managing chronic conditions prevents complications, excessive referral to subject specialists, hospital visits, the need for costly procedures and improves outcomes, resulting in reduced overall healthcare spending.^6^
- *Cost-effectiveness*: The SFPs are gatekeepers to advanced healthcare. They ensure patients receive appropriate, cost-effective care and reduce referrals and unnecessary visits to hospital specialists and emergency rooms.^7^
- *Improved treatment adherence*: Prescribed treatments are better followed when there is a consistent relationship with one SFP, leading to better health outcomes and reduced healthcare costs associated with non-adherence complications.^8^
- *Efficient resource utilisation*: Understanding a patient’s medical and care history streamlines their care and reduces duplication of tests, unnecessary procedures and administrative expenses within the healthcare system.^9^

The position article suggests solutions to critical challenges reported by SFPs working in the private sector, such as inadequate medical scheme recognition, poor communication between hospital specialists and SFPs, poorly defined roles in the policies of managed care organisations and the National Department of Health, inadequate remuneration and restricted access to specialist privileges. Some of these proposed solutions include a revised reimbursement model, which recognises SFPs as a distinct specialist group with tariffs in keeping with the complexity of managing multiple conditions. The distinctive roles of SFPs and GPs in the private sector should be better differentiated in national policies such as the National Health Insurance (NHI) policy. The SFPs should be central in coordinating multimorbid care and involved in both outpatient and inpatient care. Medical aids, other funders, hospitals and the NHI should include SFPs in their specialist staffing and encourage multidisciplinary team formation (with SFPs in a coordinating role). The SFPs are uniquely equipped to provide a diverse range of skilled services at multiple levels of care and offer capacity as specialist generalists and care coordinators in the proposed NHI programme.

The full position article is being finalised for publication on the SAAFP website. We encourage all private sector family physicians to join and help strengthen this vital forum: https://saafp.org/privatefamilyphysicianforum/.
